# Fat infiltration in the thigh muscles is associated with symptomatic spinal stenosis and reduced physical functioning in adults with achondroplasia

**DOI:** 10.1186/s13023-023-02641-5

**Published:** 2023-02-22

**Authors:** Svein O. Fredwall, Jennifer Linge, Olga de Vries, Olof Dahlqvist Leinhard, Heidi Beate Eggesbø, Harald Weedon-Fekjær, Mikael Petersson, Per Widholm, Grethe Månum, Ravi Savarirayan

**Affiliations:** 1 Sunnaas Rehabilitation Hospital, TRS National Resource Centre for Rare Disorders, 1450 Nesodden, Norway; 2 Faculty of Medicine, Institute of Clinical Medicine, University of Oslo, Oslo, Norway; 3 AMRA Medical AB, Linköping, Sweden; 4 Department of Health, Medicine and Caring Sciences, University of Linköping, Linköping, Sweden; 5 Center for Medical Image Science and Visualization, University of Linköping, Linköping, Sweden; 6 Division of Radiology and Nuclear Medicine, Oslo University Hospital, University of Oslo, Oslo, Norway; 7 Oslo Centre for Biostatistics and Epidemiology, Research Support Service, Oslo University Hospital, Oslo, Norway; 8 Department of Radiology and Department of Health, Medicine and Caring Sciences, Linköping University, Linköping, Sweden; 9 Department of Research, Sunnaas Rehabilitation Hospital, Nesodden, Norway; 10 Murdoch Children’s Research Institute and University of Melbourne, Parkville, Australia

**Keywords:** Achondroplasia, Body composition, Intramuscular fat, Magnetic resonance imaging, Muscle fat infiltration, Rehabilitation, Spinal stenosis, 6-min walk test, 30-s sit-to-stand test

## Abstract

**Background:**

Symptomatic spinal stenosis is a prevalent complication in adults with achondroplasia. Increased muscle fat infiltration (MFI) and reduced thigh muscle volumes have also been reported, but the pathophysiology is poorly understood. We explored whether the increased MFI and reduced thigh muscle volumes were associated with the presence of symptomatic spinal stenosis and physical functioning.

**Methods:**

MFI and thigh muscle volumes were assessed by MRI in 40 adults with achondroplasia, and compared to 80 average-statured controls, matched for BMI, gender, and age. In achondroplasia participants, the six-minute walk-test (6MWT), the 30-s sit-to-stand test (30sSTS), and a questionnaire (the IPAQ) assessed physical functioning.

**Results:**

Symptomatic spinal stenosis was present in 25 of the participants (the stenosis group), while 15 did not have stenosis (the non-stenosis group). In the stenosis group, 84% (21/25) had undergone at least one spinal decompression surgery. The stenosis group had significantly higher MFI than the non-stenosis group, with an age-, gender and BMI-adjusted difference in total MFI of 3.3 percentage points (pp) (95% confidence interval [CI] 0.04 to 6.3 pp; p = 0.03). Compared to matched controls, the mean age-adjusted difference was 3.3 pp (95% CI 1.7 to 4.9 pp; p < 0.01). The non-stenosis group had MFI similar to controls (age-adjusted difference − 0.9 pp, 95% CI − 3.4 to 1.8 pp; p = 0.51). MFI was strongly correlated with the 6MWT (r = − 0.81, − 0.83, and − 0.86; all p-values < 0.01), and moderately correlated with the 30sSTS (r = − 0.56, − 0.57, and − 0.59; all p-values < 0.01). There were no significant differences in muscle volumes or physical activity level between the stenosis group and the non-stenosis group.

**Conclusion:**

Increased MFI in the thigh muscles was associated with the presence of symptomatic spinal stenosis, reduced functional walking capacity, and reduced lower limb muscle strength. The causality between spinal stenosis, accumulation of thigh MFI, and surgical outcomes need further study. We have demonstrated that MRI might serve as an objective muscle biomarker in future achondroplasia studies, in addition to functional outcome measures. The method could potentially aid in optimizing the timing of spinal decompression surgery and in planning of post-surgery rehabilitation.

## Introduction

Achondroplasia is the most common skeletal dysplasia with disproportionate short stature, affecting more than 360,000 people worldwide [1, 2]. The condition is caused by a gain-of-function mutation in the fibroblast growth factor receptor 3 (*FGFR3*) gene, negatively affecting bone growth and differentiation [3, 4]. Characteristic clinical manifestations, in addition to the short stature, include rhizomelic short arms and legs, macrocephaly with frontal bossing, and midface hypoplasia [3, 5]. Several medical complications can occur throughout lifetime, including foramen magnum stenosis in infancy, central or obstructive sleep apnea, spine deformities, symptomatic spinal stenosis, tibial bowing, recurrent otitis media, conductive hearing loss, and chronic pain [2, 5–9]. In children, motor developmental milestones are delayed, while the cognitive development usually is within average range [2, 7, 10].

The spinal canal is congenitally narrow in all people with achondroplasia, due to the short pedicles and reduced interpedicular distance [4, 11, 12]. The narrow canal, combined with thoracolumbar kyphosis, lumbar hyperlordosis, and acquired age-related degenerative changes, give a high lifetime risk in achondroplasia for developing symptomatic spinal stenosis [4, 11, 13–15]. Symptoms can present already in childhood, and the prevalence increases with age [9, 16]. More than half of persons with achondroplasia will develop symptoms of spinal stenosis before the age of 40 years, increasing to over 70% during the following 10–15 years [13, 14, 17–19].

Magnetic resonance imaging (MRI) is currently regarded as the reference standard for body composition analyses, and enables an accurate and quantitative assessment of muscle fat content and fat-free muscle volumes [20–25]. In a recent MRI-based study on body composition in achondroplasia, we found increased muscle fat infiltration (MFI) and reduced fat-free thigh muscle volumes in adult participants with achondroplasia compared to matched average stature controls [26]. Sims et al. have reported similar findings in 10 young adult men with achondroplasia [27]. Studies on average stature populations have found that accumulation of MFI in the thighs occurs after only a few weeks of immobilization or physical inactivity [22, 28, 29] Following a spinal cord injury, a 30–60% reduction in muscle mass has been reported [30]. However, the association between muscle fat content, or muscle mass, and the presence of spinal stenosis has not previously been studied in patients with achondroplasia.

In the present study, we explored whether the increased MFI and reduced thigh muscle volumes were associated with the presence of symptomatic spinal stenosis and physical functioning in a Norwegian cohort of adults with achondroplasia.

## Materials and methods

### Study design, population and data collection

This cross-sectional study was part of The Norwegian Adult Achondroplasia Study, a population-based study conducted between 2017 and 2019 on community-dwelling adults living in Norway, aged 16 years or older. The recruitment process, and inclusion and exclusion criteria, have been described in detail elsewhere [17].

### Anthropometry and definition of symptomatic spinal stenosis

Anthropometric measurements and data regarding the presence of symptomatic spinal stenosis were collected from The Norwegian Adult Achondroplasia Study [17]. Symptomatic spinal stenosis was defined as the presence of, or history of, characteristic clinical symptoms of spinal stenosis, *combined* with spinal stenosis described at the correlating spine level(s) in the MRI reports, and in the surgical records for those who had undergone spine decompression surgery [17]. To confirm the presence of spinal stenosis in symptomatic non-operated participants, the MRIs were collected and re-interpreted by an experienced radiologist. In those patients, a cross-sectional anteroposterior spinal canal diameter ≤ 10 mm at minimum one spine level was considered diagnostic of symptomatic spinal stenosis [17, 31–34].

### Assessment of muscle fat infiltration and fat-free muscle volume

The assessment of MFI and fat-free muscle volumes in the thighs was performed using the MRI-based methodology developed by AMRA Medical AB (Linköping, Sweden) [20, 23, 35]. This method has been used and validated in several studies [20, 23, 35, 36], including the large UK Biobank Imaging Study with more than 10,000 participants [37, 38]. The method is further detailed in Borga et al. and Linge et al. [23, 37]. We used a 3 T MRI scanner (Discovery 750, GE Healthcare) with a 32 Channel Body Array Coil. Two sequences were used; LAVA flex (3D imaging) and IDEAL IQ sequence. The scan area was from the neck down to the ankle, with a total scanning time of six minutes [26].

The body composition analyses were performed using the AMRA Researcher software (AMRA Medical AB, Linköping, Sweden) [20, 23, 35]. The MRI scans were analyzed for MFI (in percent) and fat-free muscle volumes (in liters) in the anterior, posterior, and both (total) thigh muscle compartments. Following the automated segmentation and analysis process, an experienced operator reviewed each segmentation for anatomical correctness and technical quality. In two participants, MFI and muscle volumes could only be analyzed in one leg due to technical issues. In these two cases, we reported the value from the non-missing thigh as representative for both legs.

### Body composition profile and control group

A body composition profile was made for each participant completing the MRI scan, and for the total achondroplasia study population, based on the same methodology used in the UK Biobank Imaging Study [23]. In addition, participants with achondroplasia were compared to average stature controls (1:2) from the UK Biobank database (n = 9604), matched for body mass index (BMI), gender, and age [26]. Sitting height was used instead of height as a standardization variable in the body composition profile plots for visceral adipose tissue and total abdominal adipose tissue [26].

### Physical functioning

Data on physical functioning and activity level were collected from The Physical Fitness Study, conducted on the same study population as The Norwegian Adult Achondroplasia Study. The selection and feasibility of the physical functioning tests are further detailed in de Vries et al. [17, 39]. The six-minute walk test (6MWT) was used to assess functional walking capacity, and the 30-s sit-to-stand test (30sSTS) was used to assess muscle strength in the lower limbs [40, 41]. These tests reflect physical performances typically required in daily life situations [41–44]. The 6MWT was conducted according to the American Thoracic Society Statement guidelines [42], and the 30sSTS was conducted according to Jones et al. [40]. We recorded the total walking distance up to the nearest meter and the number of full stands.

The International Physical Activity Questionnaire short form (IPAQ) was used to assess physical activity level in adult participants with achondroplasia [39]. The participants were asked to report all their activities during the last week, specified by intensity (type of activity), duration (in minutes) and frequency. The physical activity level for each participant was then calculated in MET scores (metabolic equivalent task in minutes per week) according to the IPAQ scoring manual [45], as further detailed in de Vries et al. [39].

### Statistical analysis

For continuous variables, descriptive statistics are presented as means with standard deviation (SD). Group differences are presented with 95% independent samples t-tests confidence intervals (CI) and p-values. In participants with achondroplasia, the stenosis group was older and had higher BMI than the non-stenosis group. For comparison of MFI and muscle volumes between the stenosis group and non-stenosis group, we applied linear regression analyses, adjusting for age, gender and BMI. The adjusted difference in total MFI was evaluated by estimated marginal means. In comparison between achondroplasia participants and UK Biobank controls, perfect matching by age was not possible. Linear mixed effects regression analyses were applied to adjust for the age differences, taking into account the variation in observed levels across different matched pairs [26]. Continuity corrected chi-squared tests were used for comparing proportions (using the “prop. test” R function). Correlations was given with CI intervals based on 10,000 percentile bootstrap replications, and Pearson chi-square test p-values. Statistical analysis was performed using R version 3.6.0 (The R Foundation, Vienna, Austria) and the SPSS version 26 (IBM Corp., Armonk, New York). For all tests, statistical significance was set to p < 0.05 (two-sided).

## Results

### The achondroplasia study population

**Fig. 1:**
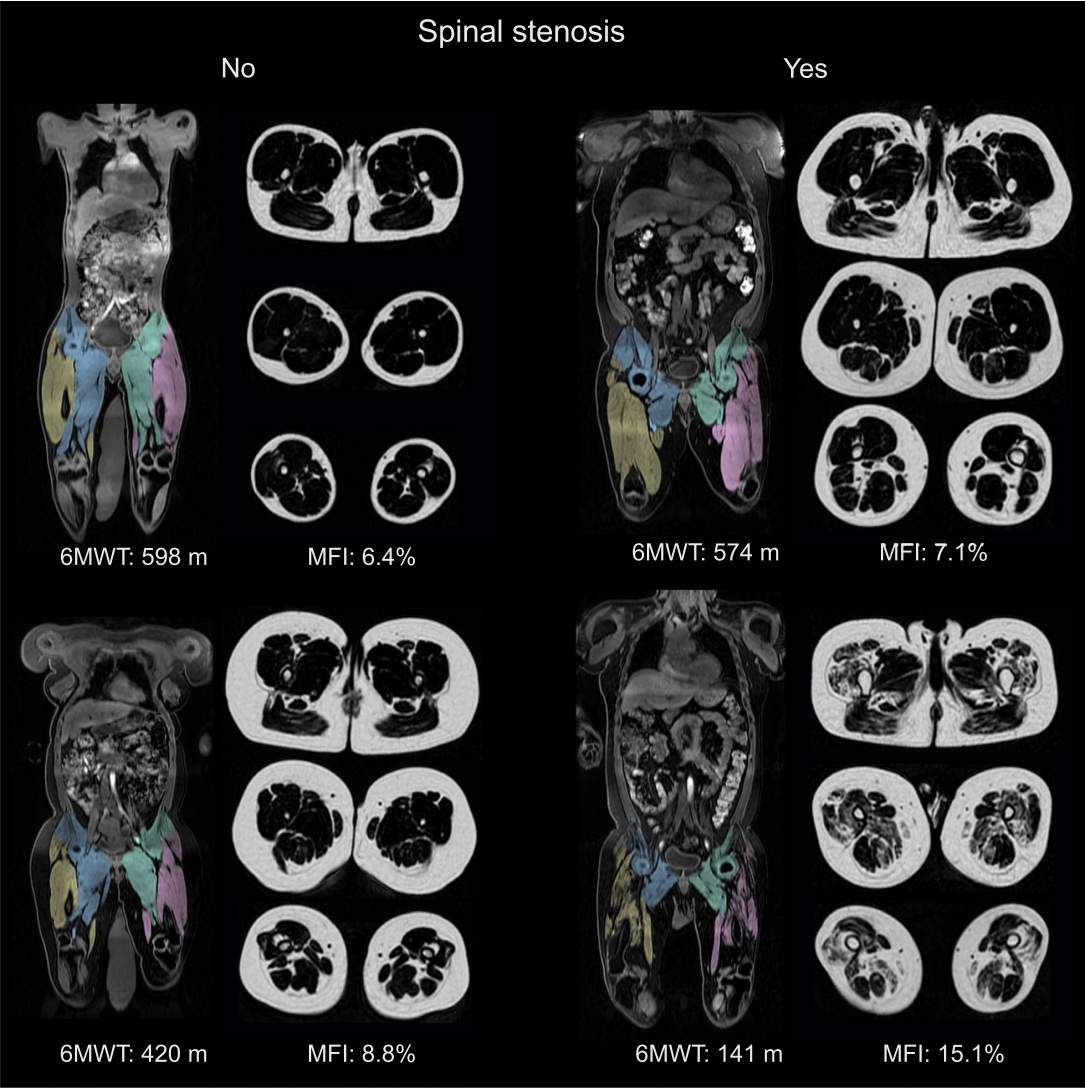
Examples of coronal and transverse magnetic resonance images in four adult participants with achondroplasia: two participants without spinal stenosis (to the left), and two participants with symptomatic spinal stenosis (to the right). The performance in the six-minute walk test (6MWT) and muscle fat infiltration (MFI) in the anterior compartment are provided for each of the participants. Muscle compartments are displayed in color

Forty participants with achondroplasia, 20 men and 20 women, completed MRI for body composition analysis (Fig. 1). Median age was 32 years (range 16–69 years). All participants had genetically confirmed achondroplasia [17]. Symptomatic spinal stenosis was present in 25 of the participants (the stenosis group), while 15 did not have stenosis (the non-stenosis group). The stenosis group was older and had higher BMI than the non-stenosis group (Table 1). Mean age difference was 20.1 years (95% CI 12.4 to 27.9 years; p < 0.01), and mean BMI difference was 5.9 kg/m^2^ (95% CI 2.1 to 9.8 kg/m^2^; p < 0.01). There were no considerable differences between the two groups regarding height, sitting height, gender, or the proportion having had lower limb extension surgery (Table 1).

**Table 1:** Characteristics of study participants with achondroplasia (n = 40)

Variables	Spinal stenosis (n = 25)	No stenosis (n = 15)	Mean difference (95% CI)	P value
	Mean (SD)	Mean (SD)		
Age, years	44.5 (16.2)	24.4 (7.8)	20.1 (12.4 to 27.9)	< 0.01
Body mass index, kg/m^2^	35.5 (6.2)	29.6 (5.2)	5.9 (2.1 to 9.8)	< 0.01
Height, cm	135.2 (9.6)	132.5 (7.4)	2.8 ( − 3.1 to 8.6)	0.35
Sitting height, cm	86.9 (4.5)	86.4 (2.7)	0.5 ( − 1.9 to 2.8)	0.68
6MWT, m ^a^	392 (123)	523 (61)	− 131 ( − 192 to − 70)	< 0.01
30sSTS, stands ^b^	20.8 (5.6)	26.6 (4.2)	− 5.7 ( − 9.2 to − 2.2)	< 0.01
Activity level, MET ^c^	2284 (2275)	3678 (2825)	− 1395 ( − 3111 to 321)	0.11
	**% (n)**	**% (n)**		
Male gender	48 (12)	53 (8)	− 5 ( − 43 to 32)	1.0
Lower limb extension, yes	28 (7)	20 (3)	8 ( − 24 to 40)	0.85
Wheelchair users	56 (14)	27 (4)	29 ( − 5 to 64)	0.14

^a^Six-minute walk test (in metres); n = 38 (stenosis group: n = 24; non-stenosis group: n = 14)

^b^30-second sit-to-stand test (number of full stands); n = 38 (stenosis group: n = 24; non-stenosis group: n = 14)

^c^MET: metabolic equivalent; n = 37 (stenosis group: n = 23; non-stenosis group: n = 14)

In the stenosis group, 96% (24/25) of the participants had lumbar spinal stenosis. In addition, 49% (10/25) had thoracic spinal stenosis, and 36% (9/25) had cervical stenosis. Age at first symptom onset varied from 11 to 57 years. Spinal decompression surgery had been performed in 84% (21/25) of the stenosis group; 13 had undergone one decompression surgery, five had two operations, and three participants had three operations. Time from symptom onset to first surgery varied from three months to 35 years, with a median of 7 years.

Thirty-eight participants (all except one permanent wheelchair user and one participant temporarily unable to perform the tests due to recent lower limb surgery) completed the physical functioning tests (the 6MWT and 30sSTS), and 37 completed the IPAQ. Participants in the stenosis group had significantly shorter walking distance than those in the non-stenosis group (392 m vs. 523 m), and performed 5.7 less stands in the 30sSTS (Table 1). The self-reported physical activity level (the IPAQ) was lower in the stenosis group than in the non-stenosis group, but the difference was not statistically significant (Table 1).

### Muscle fat infiltration and fat-free muscle volumes in the stenosis group versus the non-stenosis group

**Fig. 2:**
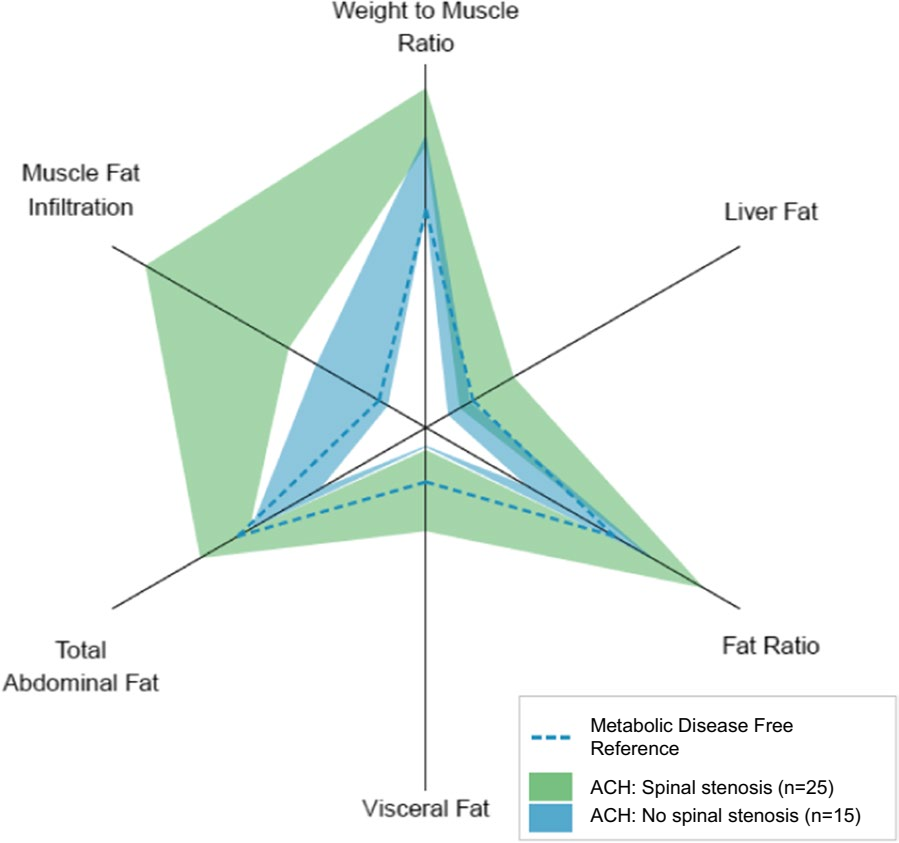
Body composition profiles in participants with achondroplasia in the spinal stenosis group (in green) versus the non-stenosis group (in blue). Dashed blue lines are reference-values based on median of the metabolic disease-free UK Biobank reference population (n = 2927)

MFI in the anterior and posterior thigh muscles compartments, and total thigh MFI, were significantly higher in the stenosis group than in the non-stenosis group (Table 2 and Fig. 2). Mean (SD) total MFI was 13.4% (4.4) in the stenosis group versus 8.2% (1.7) in the non-stenosis group. Age-, gender and BMI-adjusted difference in total thigh MFI was 3.3 percentage points (pp) (95% CI 0.4 to 6.3 pp; p = 0.03), consistent with an estimated marginal means percentwise difference of 34.6%. The observed differences in MFI between the stenosis group and the non-stenosis group in the total study population remained also in the youngest participants aged 30 years or younger, with a mean difference in total MFI of 3.6 pp (95% CI 1.0 to 6.3 pp; p = 0.01) (Table 2).

**Table 2:** Muscle fat infiltration (MFI) and fat-free thigh muscle volumes (FFMV) in the stenosis group versus the non-stenosis group in the total achondroplasia study population, and in the youngest participants (age < 31 years)

Muscle variables	Stenosis	Non-stenosis	Unadjusted		Adjusted for age, gender and BMI ^a^	
	(n = 25)	(n = 15)				
	Mean (SD)	Mean (SD)	Diff. (95% CI)	P value	Diff. (95% CI)	P value
MFI anterior, %	12.2 (5.2)	7.2 (1.4)	5.0 (2.7 to 7.2)	< 0.01	3.2 ( − 0.3 to 6.7)	0.07
MFI posterior, %	14.0 (4.4)	8.6 (1.9)	5.5 (3.4 to 7.5)	< 0.01	3.4 (0.5 to 6.3)	0.02
MFI total, %	13.4 (4.4)	8.2 (1.7)	5.3 (3.3 to 7.3)	< 0.01	3.3 (0.4 to 6.3)	0.03
FFMV anterior, L	1.8 (0.7)	1.9 (0.4)	− 0.2 ( − 0.5 to 0.2)	0.35	− 0.3 ( − 0.8 to 0.2)	0.23
FFMV posterior, L	4.2 (1.3)	4.4 (0.8)	− 0.2 ( − 0.9 to 0.4)	0.49	− 0.6 ( − 1.4 to 0.3)	0.17
FFMV Total, L	6.0 (1.9)	6.4 (1.2)	− 0.4 ( − 1.4 to 0.6)	0.43	− 0.9 ( − 2.1 to 0.4)	0.14

	Stenosis	Non-stenosis	Unadjusted	
Age < 31 years	(n = 7)	(n = 12)	Diff. (95% CI)	P value
Age, years	23.6 (5.1)	21.4 (4.4)	2.3 ( − 2.8 to 7.3)	0.34
BMI, kg/m^2^	33.6 (7.1)	29.5 (5.8)	4.1 ( − 2.9 to 11.1)	0.22
MFI anterior, %	10.4 (2.8)	7.0 (1.5)	3.4 (0.7 to 6.1)	0.02
MFI posterior, %	12.2 (3.2)	8.4 (2.0)	3.8 (0.7 to 6.9)	0.02
MFI total, %	11.6 (2.8)	8.0 (1.8)	3.6 (1.0 to 6.3)	0.01

^a^Adjusted differences estimated by linear regression

There were no considerable differences in unadjusted fat-free thigh muscle volumes between the stenosis group and the non-stenosis group (Table 2 and Fig. 2). After adjusting for age, gender and BMI, the difference in total muscle volume was -0.9 L (95% CI -2.1 to 0.4 L; p = 0.14), although not statistically significant (Table 2).

### Muscle fat infiltration and fat-free muscle volumes in participants with achondroplasia versus matched controls

**Fig. 3:**
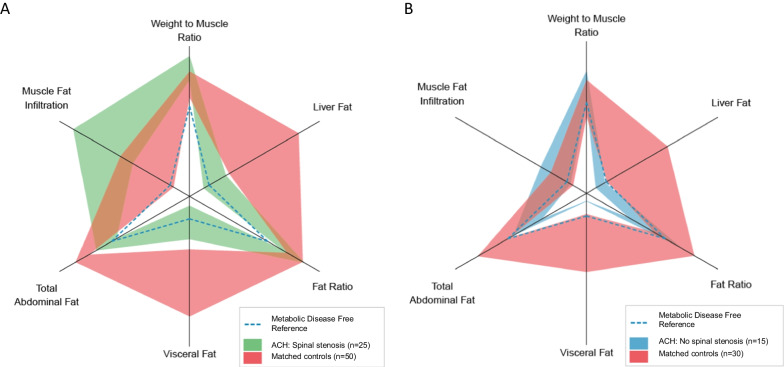
Body composition profiles in participants with achondroplasia in the stenosis group (**A**) and the non-stenosis group (**B**) compared to matched UK Biobank controls. Dashed blue lines are reference-values based on median of the metabolic disease-free UK Biobank reference population (n = 2927)

In the stenosis group, mean MFI in the thigh muscles compartments were significantly higher in participants with achondroplasia versus matched UK Biobank controls for both the anterior and posterior compartments, and for total MFI (Table 3). Mean age-adjusted difference in total thigh MFI was 3.3 pp (95% CI 1.7 to 4.9 pp; p < 0.01). In contrast, mean MFI values for the non-stenosis group were similar to UK Biobank controls, with an age-adjusted mean difference in total MFI of − 0.9 pp (95% CI − 3.4 to 1.8 pp; p = 0.51) (Table 3 and Fig. 3).

**Table 3:** Muscle fat infiltration (MFI) and fat-free muscle volumes (FFMV) in the thighs in participants with achondroplasia (ACH) compared to UK Biobank controls, matched for BMI, gender, and (partly) for age

Stenosis group	ACH (n = 25)	Controls (n = 50)	Unadjusted	Adjusted for age^a^	
	Mean (SD)	Mean (SD)	Difference (95% CI)	Difference (95% CI)	P value
Age, years	44.5 (16.2)	54.4 (6.7)	− 9.8 ( − 13.1 to − 6.5)		
BMI, kg/m^2^	35.5 (6.2)	34.5 (5.0)	1.0 (0.2 to 1.9)	1.7 (0.7 to 2.8)	< 0.01
MFI anterior, %	12.2 (5.2)	8.3 (2.5)	3.9 (2.3 to 5.5)	4.7 (2.9 to 6.4)	< 0.01
MFI posterior, %	14.0 (4.4)	12.4 (3.0)	1.7 (0.1 to 3.2)	2.6 (0.9 to 4.2)	< 0.01
MFI total, %	13.4 (4.4)	10.9 (2.8)	2.5 (1.0 to 4.0)	3.3 (1.7 to 4.9)	< 0.01
FFMV anterior, L	1.8 (0.7)	4.1 (1.1)	− 2.3 ( − 2.7 to − 2.0)	− 2.3 ( − 2.7 to − 1.9)	< 0.01
FFMV posterior, L	4.2 (1.3)	7.7 (1.7)	− 3.5 ( − 4.1 to − 3.0)	− 3.4 ( − 4.0 to − 2.7)	< 0.01
FFMV Total, L	6.0 (1.9)	11.9 (2.7)	− 5.9 ( − 6.7 to − 5.0)	− 5.6 ( − 6.6 to − 4.6)	< 0.01

Non-stenosis group	ACH (n = 15)	Controls (n = 30)	Unadjusted	Adjusted for age ^a^	
Age, years	24.4 (7.8)	50.2 (2.4)	− 25.8 ( − 28.9 to − 22.8)		
BMI, kg/m^2^	29.6 (5.2)	29.4 (4.8)	0.1 ( − 0.3 to 0.6)	0.1 ( − 1.2 to 1.4)	0.89
MFI anterior, %	7.2 (1.4)	7.0 (1.5)	0.2 ( − 0.4 to 0.8)	0.9 ( − 0.9 to 2.8)	0.35
MFI posterior, %	8.6 (1.9)	10.5 (2.3)	− 1.9 ( − 3.0 to − 0.9)	− 1.9 ( − 4.9 to 1.2)	0.22
MFI total, %	8.2 (1.7)	9.3 (2.0)	− 1.1 ( − 2.0 to − 0.2)	− 0.9 ( − 3.4 to 1.8)	0.51
FFMV anterior, L	1.9 (0.4)	4.0 (0.8)	− 2.1 ( − 2.4 to − 1.8)	− 1.4 ( − 2.3 to − 0.6)	< 0.01
FFMV posterior, L	4.4 (0.8)	7.5 (1.6)	− 3.0 ( − 3.5 to − 2.5)	− 1.4 ( − 2.7 to − 0.1)	0.04
FFMV total, L	6.4 (1.2)	11.5 (2.4)	− 5.1 ( − 5.9 to − 4.3)	− 2.7 ( − 4.8 to − 0.8)	< 0.01

^a^Adjusted differences estimated by linear mixed effects models (taking into account the variations across different matched pairs)

### Correlations between MFI and muscle volumes, and physical functioning and activity level

**Fig. 4:**
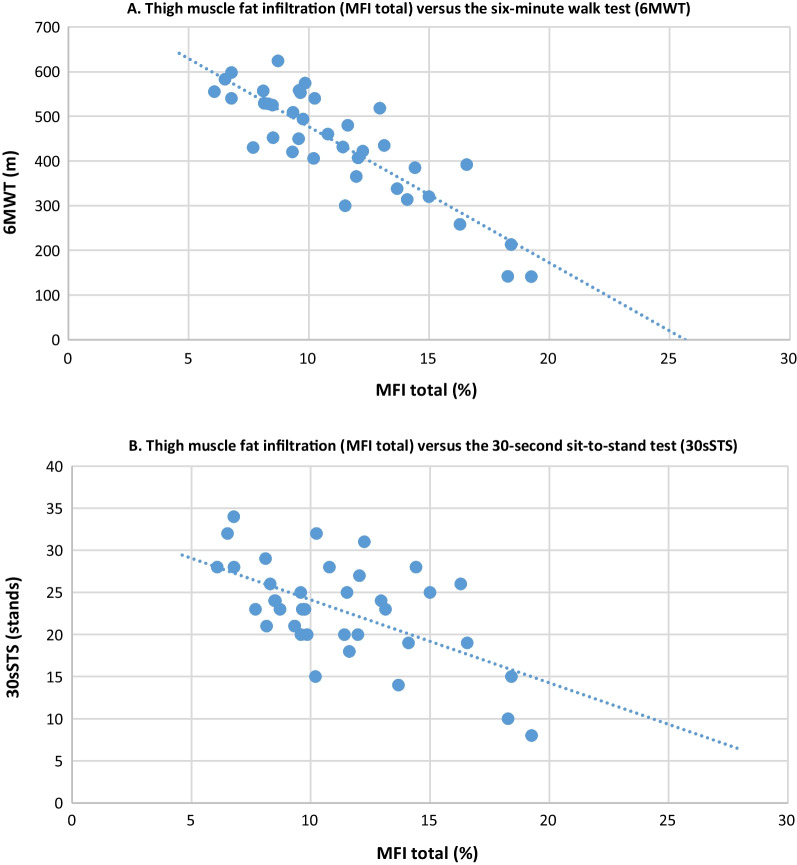
Scatter plots with trend lines displaying the correlations between total muscle fat infiltration (MFI total) in the thigh muscle compartments and the physical functioning tests in participants with achondroplasia

In participants with achondroplasia, there was a strong negative correlation between MFI and the 6MWT for both the anterior, posterior, and total MFI (r = − 0.81, − 0.83, and − 0.86; all p-values < 0.01), meaning that higher muscle fat content was correlated with shorter walking distance (Table 4 and Fig. 4). Consistently, there was a moderate negative correlation between MFI and the 30sSTS (r = − 0.56, − 0.57, and − 0.59; all p-values < 0.01), meaning that higher MFI was correlated with lower muscle strength in the lower limbs (Table 4 and Fig. 4). The 6MWT and the 30STS were positively correlated with each other (r = 0.59; p < 0.01).

**Table 4:** Correlations (with 95% confidence interval) between muscle variables and physical functioning in adults with achondroplasia (n = 38)

Variables	6MWT	30sSTS
MFI anterior	− 0.81 ( − 0.89 to − 0.68)	− 0.56 ( − 0.71 to − 0.20)
	(p < 0.01)	(p < 0.01)
MFI posterior	− 0.83 ( − 0.92 to − 0.70)	− 0.57 ( − 0.77 to − 0.25)
	(p < 0.01)	(p < 0.01)
MFI total	− 0.86 ( − 0.93 to − 0.73)	− 0.59 ( − 0.78 to − 0.25)
	(p < 0.01)	(p < 0.01)
FFMV anterior	0.36 (0.03 to 0.62)	0.01 ( − 0.32 to 0.31)
	(p = 0.03)	(p = 0.97)
FFMV posterior	0.30 (0.03 to 0.53)	− 0.06 ( − 0.33 to 0.21)
	(p = 0.06)	(p = 0.72)
FFMV total	0.33 (0.03 to 0.56)	− 0.04 ( − 0.33 to 0.24)
	(p = 0.04)	(p = 0.83)

*FFMV*: fat-free muscle volume in the thigh compartments, *MFI*: muscle fat infiltration in the thigh compartments, *6MWT*: six-minute walk test, *30sSTS*: 30-s sit-to-stand test

## Discussion

In this study, we explored potential associations between MFI and fat-free muscle volumes in the thigh muscles, and the presence of symptomatic spinal stenosis and physical functioning in a cohort of Norwegian adults with achondroplasia. Participants in the stenosis group had significantly higher MFI than the non-stenosis group and healthy average stature controls, while the non-stenosis group had low MFI, and similar to healthy controls. In contrast, there were no significant differences in thigh muscle volumes between the stenosis group and the non-stenosis group. There was a moderate to strong correlation between MFI and the physical functioning tests.

To our knowledge, this is the first study investigating muscle fat content and muscle volumes in achondroplasia by using MRI. Sims et al. have previously reported increased fat mass in the thighs in 10 adult men with achondroplasia, as assessed by Dual Energy X-Ray Absorptiometry (DXA) [46]. However, DXA is unable to quantify muscle fat distribution or assess muscle quality [25]. Nor did the study by Sims and colleagues provide information about the presence of spinal stenosis in their participants.

Studies on average stature populations have demonstrated a substantial change in the skeletal muscles below the level of injury after a spinal cord injury [22, 28, 30, 47]. This also includes patients with an incomplete spinal cord injury [22, 28, 30, 47]. In a study of patients with an acute incomplete spinal cord injury, the intramuscular fat accumulation in the thighs was 26% higher, and the skeletal muscle volume 33% lower, six weeks post-injury compared to baseline [47]. Moreover, the muscle fat content increased with an additional 26% during the next three months [47]. Consistently, in our study, adjusted total thigh MFI was about 35% higher in the spinal stenosis group as compared to the non-stenosis group.

Notably, also the youngest achondroplasia participants in the stenosis group had increased thigh MFI, while participants in the non-stenosis group of approximately same age had low MFI, and similar to healthy controls. Overall, our data suggest that the increased MFI observed in participants with achondroplasia is related to the presence of spinal stenosis, consistent with the accumulation of MFI in the thigh muscles observed in patients following a spinal cord injury.

While we observed the same pattern of increased MFI in the stenosis group as reported in patients with a spinal cord injury, there were no significant differences in muscle volumes between the stenosis group and the non-stenosis group. All achondroplasia participants had decreased thigh muscle volumes compared to average stature controls. This might be explained by the shorter femur length in achondroplasia, as also suggested by Sims et al. [46]. This is also consistent with studies conducted on average stature populations, having displayed that a small body size was associated with low muscle quantity [38].

The difference in age and BMI between the stenosis group and the non-stenosis group are potential confounding factors in our study, as MFI tends to increase with age and higher BMI [21, 38]. This was demonstrated in the UK Biobank Imaging Study, where 9615 participants underwent muscle MRI. MFI was negatively correlated with age and with an increase in fat content of 0.4 pp per 5-year [38]. We therefore adjusted for age and BMI. However, while the observed unadjusted differences in total MFI of 5.3 pp in our study was reduced by age and BMI adjustment, the difference was still of 3.3 pp, supporting that there is an actual difference. We also adjusted for gender in the regression analyses, but this did not affect the outcome.

In studies on average stature populations, increased MFI is associated with decreased six-minute walking distance, decreased gait speed, decreased physical performance, and difficulty with repeated chair stands [21, 22, 30, 48, 49]. Increased MFI is also a predictor of future mobility limitations [22, 50, 51]. In a study conducted on average-statured older adults followed for 2.5 years, persons with increased MFI were 50–80% more likely to develop mobility limitations compared to those with the lowest MFI levels at baseline [22, 50]. Consistently, in our study, increased MFI was correlated with shorter walking distance and reduced muscle strength in the lower limbs. Moreover, there was a positive correlation (r = 0.59) between the 6MWT and the 30sSTS in achondroplasia, consistent with studies conducted in other medical conditions [52, 53].

In the UK Biobank Imaging Study, an adverse muscle composition (defined as increased MFI and reduced thigh muscle volumes) was a strong predictor of all-cause mortality [54]. Isolated increased MFI was associated with an all-cause mortality hazard-ratio similar to a previous cancer diagnosis or smoking. The combination of adverse muscle composition *and* poor physical functioning (as assessed by hand grip strength and self-reported walking capacity) identified the most vulnerable patients in the UK Biobank study [54]. Interestingly, in a prior study conducted in the US, an increased mortality and a 10-year shorter life expectancy have been reported in adults with achondroplasia [55]. The causes for the increased mortality are not fully understood, but neurological complications were among the most frequently reported causes of death, in addition to heart disease and accidents [55].

In studies conducted on average stature populations, reduced physical activity is associated with increased MFI [22, 28, 29]. The cross-sectional design of our study preclude the possibility of drawing conclusions on causality. However, in a previously published study on the same study population, we found that the presence of symptomatic spinal stenosis was associated with increased pain intensity and frequency and reduced mobility, factors that might explain the increased MFI observed in the spinal stenosis group [17]. Consistent with this, in the present study, the non-stenosis group reported higher mean physical activity level (in MET) in the last week than the stenosis group, but the difference did not reach statistically significance in our limited sized study population. In addition, there was a considerable variability in the reported physical activity level within the achondroplasia study population, both in the stenosis group and the non-stenosis group [39].

### Strengths and limitations

Strengths of this study are the objective muscle MRI measurements combined with the assessment of physical functioning. Genetically confirmed achondroplasia in all participants, comparison with a matched control group, and symptomatic spinal stenosis defined by clinical symptoms and verified by medical records *and* images are other strengths.

There are also several limitations to this study, including a relatively small sample size and the cross-sectional study design. The difference in age and BMI between the stenosis group and the non-stenosis group, and the age difference between participants with achondroplasia and the matched controls, might have affected the outcome, although we adjusted for linear effects in the statistical analyses. Further study is required to validate our findings, and preferably with a longitudinal study design.

### Clinical implications and further research

Muscle fat infiltration (MFI) is a muscle specific measurement indicating quality of the muscle, and has been shown to have a strong correlation to functional performance, such as the 6MWT [56]. However, the functional performance can be confounded by the individual’s motivation, cooperation and fitness level, as well as by day-to-day variation and pain [38, 56] Quantitative MRI is independent of patient performance, is more sensitive to early disease progression, and with less individual variability [56]. Moreover, individuals that progress in their disease may eventually become unable to complete a functional performance test, while muscle MRI can be carried out even when function is lost [56]. In studies on several neuromuscular conditions, MRI has demonstrated a strong correlation with functional performance tests, such as the 6MWT [20, 56]. We have demonstrated that this also applies to people with achondroplasia.

Currently, surgery is the only curative treatment option for progressive symptomatic spinal stenosis in achondroplasia [4, 57]. However, there is no general agreement on the optimal timing for surgical intervention, although some studies have suggested a more favorable outcome if an early surgical intervention is performed after onset of symptoms [11, 12]. Data from our study demonstrate a great variability both in age at first symptom onset (varying from 11 to 57 years) and time from symptom onset to first surgery (varying from three months to 35 years). However, whether an earlier surgical intervention would translate into higher physical performance and less accumulation of MFI in patients with achondroplasia, presenting with symptoms of spinal stenosis, require further study, preferably with a prospective longitudinal design.

Moreover, studies on average stature populations have demonstrated that MFI can be reversible, at least partially, by early and structured resistance exercise [28, 58]. What effects a structured rehabilitation program might have on functional outcomes and MFI in patients with achondroplasia, remains to be studied.

## Conclusion

In this cohort of Norwegian adults with achondroplasia, increased MFI in the thigh muscles was associated with the presence of symptomatic spinal stenosis, reduced functional walking capacity, and reduced lower limb muscle strength. The increased thigh MFI observed in participants with achondroplasia with spinal stenosis is consistent with the accumulation of MFI in the thigh muscles observed in patients in the general population following a spinal cord injury. Further studies are needed to determine the causality between spinal stenosis and the accumulation of thigh MFI, including the optimal timing of surgical intervention. We have demonstrated that MRI might serve as an objective muscle biomarker in future achondroplasia studies, in addition to functional outcome measures. The method could potentially aid in optimizing the timing of spinal decompression surgery and in planning of post-surgery rehabilitation.

## Data Availability

De-identified individual participant data are available from the corresponding author on reasonable request.
